# Sufficient Numbers of Early Germ Cells Are Essential for Female Sex Development in Zebrafish

**DOI:** 10.1371/journal.pone.0117824

**Published:** 2015-02-13

**Authors:** Xiangyan Dai, Xia Jin, Xiaowen Chen, Jiangyan He, Zhan Yin

**Affiliations:** 1 Key Laboratory of Aquatic Biodiversity and Conservation of the Chinese Academy of Sciences, Institute of Hydrobiology, Chinese Academy of Sciences, Wuhan, Hubei, China; 2 University of Chinese Academy of Sciences, Beijing, China; National University of Singapore, SINGAPORE

## Abstract

The sex determination for zebrafish is controlled by a combination of genetic and environmental factors. The determination of sex in zebrafish has been suggested to rely on a mechanism that is affected by germ cell-derived signals. To begin our current study, a simplified and efficient germ cell-specific promoter of the *dead end* (*dnd*) gene was identified. Utilizing the metrodinazole (MTZ)/ bacterial nitroreductase (NTR) system for inducible germ cell ablation, several stable Tg (*dnd*:NTR-EGFP^-3'UTR^) and Tg (*dnd*:NTR-EGFP^+3'UTR^) zebrafish lines were then generated with the identified promoter. A thorough comparison of the expression patterns and tissue distributions of endogenous *dnd* and *ntr-egfp* transcripts *in vivo* revealed that the identified 2032-bp zebrafish *dnd* promoter can recapitulate *dnd* expression faithfully in stable transgenic zebrafish. The correlation between the levels of the germ cell-derived signals and requirement for maintaining the female fate has been also explored with different durations of the MTZ treatments. Our results revealed the decreasing ratios of female presented in the treated transgenic group are fairly associated with the reducing levels of the early germ cell-derived signals. After the juvenile transgenic fish treated with 5 mM MTZ for 20 days, all MTZ-treated transgenic fish exclusively developed into males with subfertilities. Taken together, our results identified here a simplified and efficient *dnd* promoter, and provide clear evidence indicating that it was not the presence but the sufficiency of signals derived from germ cells that is essential for female sex development in zebrafish. Our model also provides a unique system for sex control in zebrafish studies.

## Introduction

The existence of mechanisms of sex determination in teleosts is notably diverse. The genetic sex determination (GSD) system is used in mammals and in certain fish. However, in zebrafish and other fish, there is still no evidence for the existence of a sex-specific genomic system. Independent of genetic background, the early gonads in zebrafish initially develop into an ovary-like “bipotential juvenile ovary.” These “bipotential juvenile ovaries” can develop into either mature ovaries in females or into testes following oocyte apoptosis in the “juvenile ovaries” in males. However, environmental conditions, such as temperature, food and hypoxia, can affect the sexual fate of zebrafish, which is known as environmental sex determination [1,2,3]. Recently, studies on transcriptional expression manipulation have revealed the contribution of some genes in the sex determination pathway of zebrafish, such as *fancl* [4], *ftz-1* [5] and *fst-1* [6]. However, little information is available regarding the mechanisms of zebrafish sex determination.

DND, a vertebrate germ-plasma component that is specifically expressed in primordial germ cells (PGCs), has been reported as an essential factor for zebrafish PGCs survival, maintenance and migration [7]. Depletion of functional DND at embryonic stages in zebrafish by application of gene-specific morpholino (MO) caused both the complete loss of germ cells as well as the promotion of all the morphant development as sterile males [8]. This has demonstrated the important role of germ cells in the zebrafish sex determination process. A similar phenomenon occurs in medaka, a teloest model where some evidence of GSD exists. Germ cell-deficient medaka fish were generated when the *C-X-C chemokine receptor type 4* (*cxcr4*) MO was injected into early embryos, and these fish showed a female-to-male sex reversal with a single tube-like gonad expressing a high level of androgen, normal male-specific genes, and decreased levels of estrogen [9]. This suggested that the sex fate of most teleosts with a GSD system was determined by somatic cells under the control of the genetic sex chromosome rather than an interaction between the somatic cells and germ cells [10,11]. However, insights on the mechanisms determined by germ cells or the interaction between germ cells and somatic cells in zebrafish were explored under the same rearing environments.

In recent years, using PGC-depleted zebrafish, numerous chimeras were obtained using the transplantation technique. Each PGC-depleted zebrafish transplanted with a single PGC from zebrafish or pearl danio (*Danio albolineatus*) developed into a phenotypical male [12]. Subsequently, one type of sterile *Danio* hybrid was generated by *in vitro* fertilization using pearl danio’s sperm with zebrafish eggs. The transplantation of adult zebrafish ovarian germ cells into 2-week-old sterile *Danio* hybrid larvae resulted in phenotypic male and female germline chimeras. However, only some of the male germline chimeras were fertile [13]. Thus, all the zebrafish germline chimeras were re-introduced with germ cells using current techniques could still only develop into functional males.

Bacterial NTR is an enzyme that can convert the innocuous prodrug MTZ to a cytotoxic product, which induces cell death [14]. Using transgenic methods to specifically express NTR in zebrafish tissue under a tissue-specific promoter results in an inducible chemical-genetic cell ablation zebrafish hybrid [15,16]. Recently, utilizing 5-month old nanos3-null mutant and *ziwi*:CFP-NTR transgenic zebrafish, Dranow *et al*. (2013) demonstrated that adult females can sex-revert to functional males when oocytes in the mature ovary are depleted [17]. This further indicates that signals derived from the germ cell or oocyte act on the somatic gonad to regulate gonadal development.

In our present studies, a 2032-bp zebrafish *dnd* promoter was isolated and used to faithfully recapitulate *dnd* expression in stable transgenic zebrafish. At least three stable *dnd*:NTR-EGFP independent transgenic lines were generated. When these transgenic fish at 18 dpf were treated with MTZ (5 mM) for 20 days, all germ cell-depleted transgenic juveniles developed into males with low fecundity. We confirmed the apoptotic effects in NTR-EGFP-expressing germ cells. Our results further demonstrated that the signal derived from the quantity of germ cells determines the sex fate of zebrafish.

## Materials and Methods

### Zebrafish maintenance

Our zebrafish were maintained in 16 L tanks at 28.5° according to zebrafish book. Embryos were obtained by natural spawning and were cultured in egg water. All procedures related to the care and use of the fish were approved by the Ethics Committee from Institute of Hydrobiology, Chinese Academy of Sciences (Approval Protocol No.IHB20110089) according to the National Guiding Principle for the Care and Use of Laboratory Animals.

### Cloning of the zebrafish *dnd* promoter and *dnd*:EGFP-NTR transgenic vector construct

The backbone for our transgenic vector was made by introducing the amplified MCS, ires and egfp part from Pires-EGFP into PUC19 plasmid. Besides, two meganuclease I-SceI sites were introduced to improve transgenic efficiency [18]. We refer this plasmid as the *I-SceI*-MCS-*ires*-*egfp*-*I-SceI* vector.

**Fig 1:**
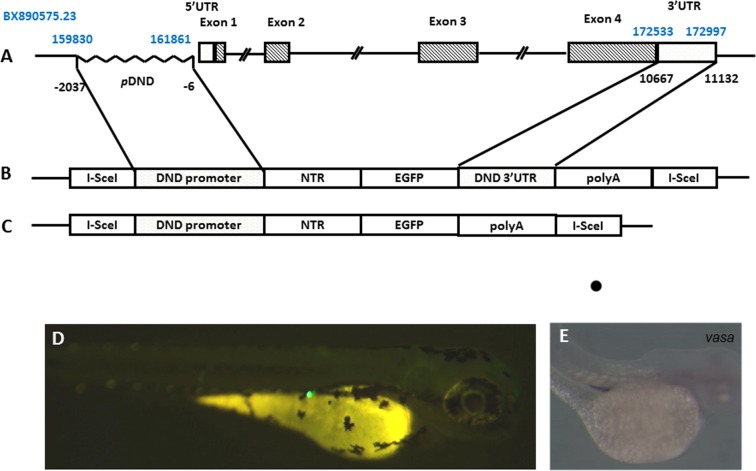
Generation of the transgenic zebrafish with the NTR-EGFP expression under the control of zebrafish *dnd* promoter. A) genomic structure of zebrafish dnd gene, including 5’UTR region, 4 exons and 3’ UTR region identified for its PGC-specific presence in the fourth exon. The positions for the region in zebrafish DNA sequence BX890575.23 are labeled in blue. The position sites for the region used related to the transcriptional starting site of *dnd* are labeled in black. B) schematic representation of the transgenic construct containing the 5’-*dnd*:*ntr-egfp-3’UTR of dnd-*3’ fragment flanked by I-Sce1 sites. C) Schematic representation of the transgenic construct containing the 5’-*dnd*:*ntr-egfp* without *dnd-*3’ fragment flanked by I-Sce1 sites. D) Fluorescence microscopic image showing EGFP-expressing PGCs of a F0 progeny at 72 hpf. E) in situ hybridization signals *of vasa* probe showing the position of PGC at 72 dpf.

According to *dnd* genomic DNA in the NCBI GenBank (Gene ID: 373074), a 2032bp fragment was amplified using the following primers (Fig. 1A): 5’-GGTCATAAACTCAGCTCAGCGA-3’ (forward primer); 5’-CCTCATGACTCCTAGAGACGCTAT-3’ (reverse primer). The amplified fragment was cloned into the *I-SceI*-MCS-*ires*-*egfp*-*I-SceI* vector. The *ires* element was then replaced with *ntr* coding region amplified from *E. coli* genomic DNA as shown before [19] to generate “*I-SceI*-dnd:NTR-EGFP-polyA-*I-SceI* vector” (for *dnd^-3’UTR^*:NTR-EGFP transgenic fish) (Fig. 1C). In addition, 466-bp of the 3’UTR of *dnd* [7] was amplified and introduced into the backbone of “*I-SceI*-dnd:NTR-EGFP-polyA-*I-SceI* vector” in front of polyA. This vector was then referred as “*I-SceI*-dnd:NTR-EGFP-3’UTR-polyA-*I-SceI* vector”(*dnd^+3’UTR^*:NTR-EGFP transgenic fish) (Fig. 1B).

### Transgenic vector microinjection and screening strategy

The transgenic vectors and I-SceI meganuclease were co-injected into 1-2-cell stage zebrafish embryos [18]. Embryos with EGFP in PGCs were identified under fluorescence microscope at 48 hours post- fertilization (hpf) (Fig. 1B and C), and raised up. After the F0 zebrafish reached sexual maturation, they were mated with wild-type zebrafish adults. Genomic DNA PCR and fluorescence microscopy were used for stable germline founder screening. F1 embryos with EGFP expression were collected, and the genomic DNA samples were prepared from the 2-dpf larvae. PCR detection on the fragment of the intact region containing the *dnd* promoter and NTR components was performed using the following primers: 5’-GAGCAAGCCACACATTTCTAAC-3’ (forward) and 5’-CGGCAGCGTAGCGTTAAAATC-3’ (reverse) to confirm the stable germline transmission of *dnd*:NTR-EGFP transgenic zebrafish.

### RT-PCR and quantitative real time PCR

Total RNA was purified from zebrafish embryos, larval and adult tissues according to the manufacturer’s instructions using Trizol Reagent (Gibco Invitrogen, Carlsbad, CA). To measure the maternal and early expression of *dnd* at 0 hpf or at 0.2 hpf (12 min post-fertilization) stage, the artificial fertilization procedure has been used. Briefly, the extrusive matured gametes were collected from the cloaca of the adult males and females separately with gentle squeezing press on the fish abdomen. The sperm are maintained in Hank's saline. Before the sperm were added, the stage for the eggs was treated as 0 hpf. Twelve minutes after the sperms were mixed with the eggs, we treated it as 0.2 hpf. Reverse transcription (RT) PCR was performed using the Reverse Aid First-stand cDNA Synthesis Kit (Fermentas, Burlington, Canada). The primers for *dnd, β-actin* and *NTR*:*EGFP* used in the RT-PCR and quantitative real-time PCR assay were: 5’-ACCCAAGTCAATGGGCAGAG (*dnd* forward), 5’-TGCCCGCTGAAGTTCATCAT (*dnd* reverse), 5’-CGAGCAGGAGATGGGAACC (*β-actin* forward), 5’-CAACGGAAACGCTCATTGC (*β-actin* reverse), 5’- ATGGATATCATTTCTGTCGCC (*NTR*:*EGFP* forword), and 5'- GACGTTGTGGCTGTTGTAGTTG (*NTR*:*EGFP* reverse).

### Germ cell ablation assay

MTZ solution (5 mM) was made by dissolving MTZ powder ((Sigma, Cat. No. 443-48-1. USA) into egg water with 0,2% DMSO added as previous study [14]. When NTR-EGFP-positive larvae were identified at 18 dpf, each group with randomly selected fifteen to twenty fish was transferred into a 2 L tank. They were treated in 500 ml of 5 mM MTZ/ 0.2% DMSO or 0.2% DMSO control solution for 20 days in a dark environment. The solutions were changed every other day. During treatment, the fish were fed twice per day with live fairy shrimp. After the 20-day treatment, all of the fish groups were raised to sexual maturation stage under normal hatchery conditions. The sex ratio and fecundability of these fish were then analyzed and recorded.

### *In situ* cell death assay

Transgenic fish at 18 dpf were immersed in 5 mM MTZ solution for one week in the dark. The larval gonads were dissected and cryosectioned at 6 μm thickness.A TUNEL assay was performed according to the instructions of In Situ Cell Death Detection Kit, tetramethylrhodamine (TMR) red (Roche Diagnostics Gmbh). And the images were recorded on flurescence microscope and analyzed using photoshop software.

### Fecundability assessments

Adult MTZ-treated males and their sibling control males were crossed with wild-type females every week for three different valid mating trials, and the fertilization rate was presented as the ratio of fertilization eggs to the total spawning eggs for each mating. Males were individually housed during each breeding trial.

### Anatomical and histological analyses

The genders of fish were determined by dissection after 70 dpf stage. Only for the fish for the fecundability assessments, the dissections were done after the mating serials. The cryo-sections of larval tissue were stained with hematoxylin and eosin. The definitions of the stages of the oocytes and spermatocytes in zebrafish gonads were following previous descriptions [4].

### Statistical analysis

The fecundability and the quantative real time datas were analyzed by nonparametric tests and student T-test analysis using SPSS. *p≤0.001* was considered significantly different.

## Results

### Identification of the *dnd* promoter and generation of two Tg(*dnd*:NTR-EGFP) zebrafish lines

**Fig 2:**
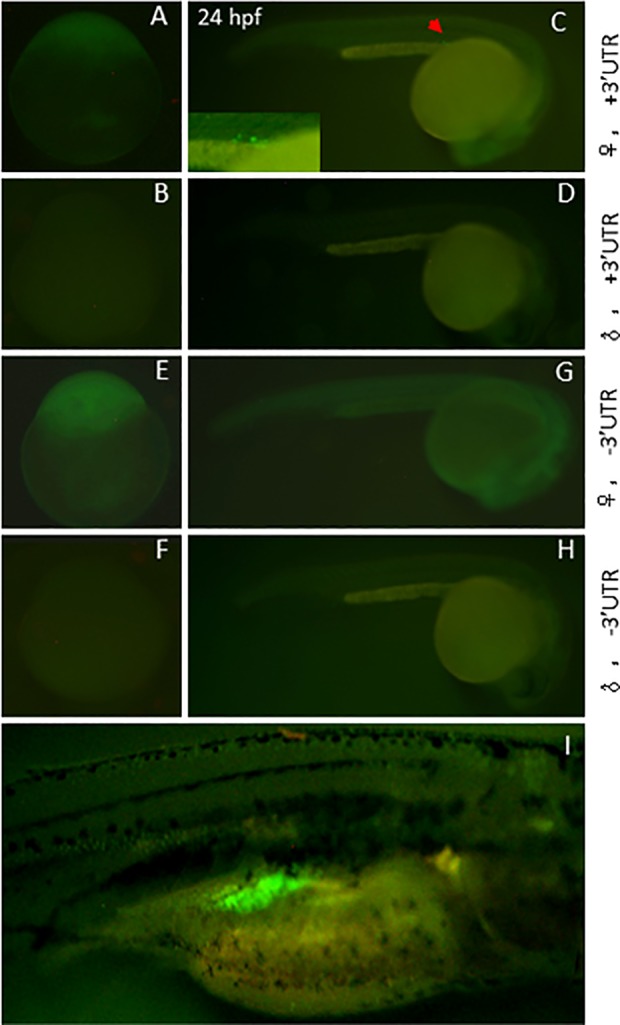
Germ line expression of the *dnd* ^+3’UTR^:NTR-EGFP and *dnd* ^-3’UTR^:NTR-EGFP transgene in zebrafish. The fluorescent protein expression was monitored using fluorescence microscopy. A-D) Expression pattern of the transgene in *dnd^+3’UTR^*:NTR-EGFP transgenic fish. A and C, the expression pattern following female transmission, with PGC-specific expression at 24 hpf stage (the inset in C shows the EGFP-positive PGCs in transgenic embryos); B and D, no fluorescence expression following male transmission. E-H) Expression pattern of the transgene in *dnd^-3’UTR^*: NTR-EGFP transgenic fish. E and G, the expression pattern following female transmission, without PGC-specific expression at 24 hpf stage (G); F and H, no fluorescence expression following male transmission either. I) early germ cell-specific expression patterns of transgene at 18 dpf in both *dnd^+3’UTR^*:NTR-EGFP or *dnd^-3’UTR^*: NTR-EGFP transgenic fish, following either female or male transmission.

**Fig 3:**
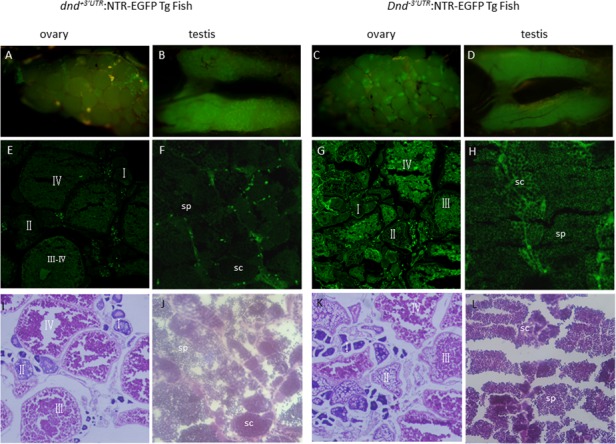
Gonadal expression of the *dnd^+3’UTR^*:NTR-EGFP and *dnd^-3’UTR^*:NTR-EGFP transgene in adult zebrafish. Anatomical views of the gonadal expression of the NTR-EGFP transgene are presented in A-D; Views of NTR-EGFP trangene expression and histological features with HE staining in the cyosectioned gonadal tissue are presented in E-H and I-L; A and B) Expression of the transgene in zebrafish ovary (A) and testis (B) in *dnd^+3’UTR^*:NTR-EGFP transgenic fish at 100 dpf; C and D) Expression of the transgene in zebrafish ovary (C) and testis (D) in *dnd^-3’UTR^*:NTR-EGFP transgenic fish at 100 dpf; E and F) NTR-EGFP transgene expression in the cryosections of the ovary (E) and testis (F) of the F1 progenies of *dnd^+3’UTR^*:NTR-EGFP transgenic fish at 100 dpf; G and H) NTR-EGFP transgene expression in the cryosections of the ovary (G) and testis (H) of the F1 progenies of *dnd^-3’UTR^*:NTR-EGFP transgenic fish at 100 dpf; I-J) histological features with HE staining for the I-J) shows the HE staining for the cryosections of the ovary (I) and testis (J) of the F1 progenies of *dnd^+3’UTR^*:NTR-EGFP transgenic fish at 100 dpf; K-L) shows the HE staining for the cryosections of the ovary (K) and testis (L) of the F1 progenies of *dnd^-3’UTR^*:NTR-EGFP transgenic fish at 100 dpf; Fluorescence was observed in the oocytes at all stages (E, G, I, K) and in spermatocytes(sc) and sperm(sp) (F, H, J, L).

To explore role of *dnd* gene in zebrafish gonadal development, our pilot experiments confirmed a 2032 bp upstream of dnd transcription start site can drive PGC specific EGFP expression (Fig. 1). Combined with NTR-MTZ mediated cell death techiques [14], In our current study, two constructs expressing NTR-EGFP driven by *dnd* promoter region with or without 3’UTR (see methods and materials) were used to generate transgenic lines. In our transient F0 larvae, this 2032-bp promoter region was sufficient to drive NTR-EGFP expression in the PGCs in approximately 150/500 injected embryos with or without the 3’UTR of *dnd*. The transient EGFP signal was restricted in PGCs at 2 dpf (Fig. 1C). These EGFP-positive F0 were raised to sexual maturity and crossed with wild-type zebrafish for screening germline transgenic fish. Interestingly maternal fluorescence protein was observed in the F1 embryos at the early stage in both Tg(*dnd^-3’UTR^*:NTR-EGFP) and Tg(*dnd^+3’UTR^*:NTR-EGFP) fish (Fig. 2A and E). The levels of maternally expressed fluorescence protein gradually decreased in these embryos in both lines, but still could be observed during the 24–48 hpf stage in Tg(*dnd^+3’UTR^*:NTR-EGFP) fish (Fig. 2C), but not in Tg(*dnd^-3’UTR^*:NTR-EGFP) line, indicating a role of *dnd* 3’UTR in early PGC development, but not for late PGC maintenance. Furthermore, expression of the NTR-EGFP in early PGCs disappeared in the F1 larvae after 48 hpf. Interestingly, the gonad-specific expression of the fluorescence protein resumed in these F1 larvae after 18 dpf in both lines (Fig. 2I). Gonad cross-section experiments assumed that the expression of the fluorescence protein continued in gonadal tissues, in both the ovary (Fig. 3A and C) and testis (Fig. 3B and D). Unexpectedly, a higher level of NTR-EGFP expression in 3 lines of Tg(*dnd^-3’UTR^*:NTR-EGFP) fish was observed than those in the 3 independent lines of Tg(*dnd^+3’UTR^*:NTR-EGFP) line (Fig. 3). These results suggest that both our lines can drive specific NTR-EGFP expression in PGCs and gonads in a time-controlled manner.

### The transgenic lines can faithfully mimic endogenous *dnd* expression

**Fig 4:**
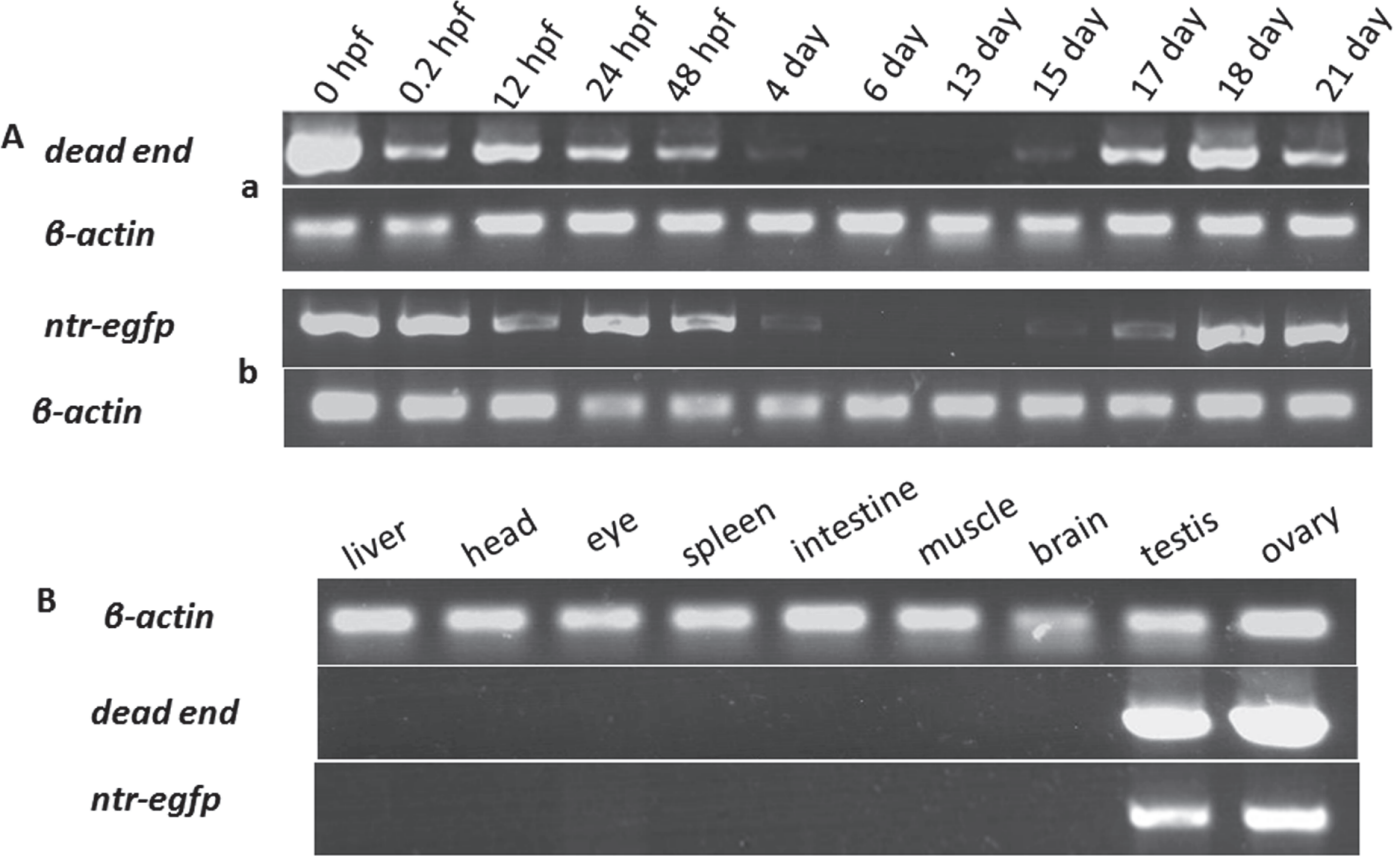
Detection of the transcripts of *dnd* and *ntr-egfp* transgene in transgenic fish at different developmental stages. Endogenous *dnd* or exogenous *ntr-egfp* transcript was detected using RT-PCR. A) endogenous *dnd* (a) or exogenous *ntr-egfp* (b) transcript detected in the fish body at different developmental stages. B) Endogenous *dnd* (middle) or exogenous *ntr-egfp* (bottom) transcript detected in various tissues from adult fish at 120 dpf. Transcripts of *β-actin* were amplified from the same samples as an internal control to test the quality of the cDNA template.

Then we compared expression patterns of NTR-EGFP in the two different transgenic lines with endogenous *dnd* to see if our transgenic lines can be used for further study. We used RT-PCR assay to check the *dnd* endogenous expression. As shown in Fig. 4A, our results indicated that zebrafish *dnd* is maternally expressed at 0-hpf stage, and then gradually decreased from 48 hpf, where little or no mRNA was detected in the developing larvae between 4–17 dpf stages. At 18-dpf, endogenous *dnd* was strongly expressed in undifferentiated ovary-like gonads of zebrafish juvenile (Fig. 4A, upper panel). After sexual maturation, *dnd* was only expressed in sex gonads, both testis and ovary (Fig. 4B, middle panel). All these results were consistent with previous study [7]. The same assay was also performed to examine the temporal patterns and tissue distribution of *ntr-egfp* expression in *dnd^+3’UTR^*:NTR-EGFP transgenic zebrafish in the early embryos and adult tissues. As shown in Fig. 4, identical expression patterns of *ntr-egfp* in our stable transgenic lines were observed (Fig. 4Ab, upper panel; Fig. 4B, bottom panel. We subsequently selected the progenies of stable transmission *dnd^-3’UTR^*:NTR-EGFP transgenic fish for the following MTZ-treatment experiments. The F2 *dnd^-3’UTR^*:NTR-EGFP transgenic fish and their non-transgenic sibling control zebrafish were used for further analyses.

### NTR-MTZ mediated technique can be used to ablate PGCs in transgenic lines

**Fig 5:**
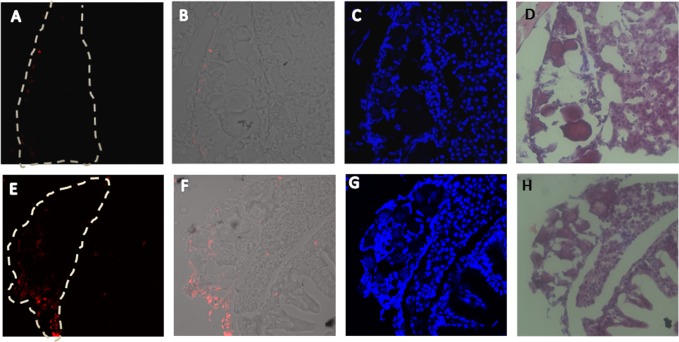
Induced cell death in transition gonads of the *dnd*:NTR-EGFP transgenic fish via MTZ treatments. The MTZ-induced cell death were evaluated by an *in situ* cell death assay in the transition gonads of the *dnd*:NTR-EGFP transgenic fish at 26 dpf stage after 1 week MTZ treatment. Transgenic fish at 18 dpf were treated with 5 mM MTZ/0.2% DMSO or 0.2% DMSO solution for 1 week. The fish were cryosectioned at 6μm for *in situ* TUNEL assay. A-D) transition gonad after MTZ treatments with apoptosis signals under fluorescent microscope (red fluorescence, A), merge images under fluorescent microscope and white light microscope (B), DAPI staining (C), HE staining (D); E-H) transition gonad in control groups with apoptosis signals under fluorescent microscope (red fluorescence, E), merge images under fluorescent microscope and white light microscope (F), DAPI staining (G), HE staining (H); The white frame indicates the gonad position.

Although several studies show that NTR-MTZ combined technique can be used for inducing specific cell death in doze of cells and tissues including zebrafish [15,16,20], few gonad specific ablation was made until now. So to confirm the suitability of this technique in zebrafish gonads, transgenic fish at 18 dpf were treated with 5 mM MTZ for one week. The gonadal tissues were dissected and cryosectioned for the TUNEL assay. As shown in Fig. 5, there was a significantly higher number of apoptotic cells detected in MTZ-treated Tg(*dnd*:NTR-EGFP) fish than those in control transgenic fish. This trial confirmed that NTR-EGFP expressed in our transgenic lines could efficiently mediate specific cell apoptosis in gonadal tissues after MTZ addition.

### Zebrafish sexual differentiation and fecundity after early germ cell loss

**Fig 6:**
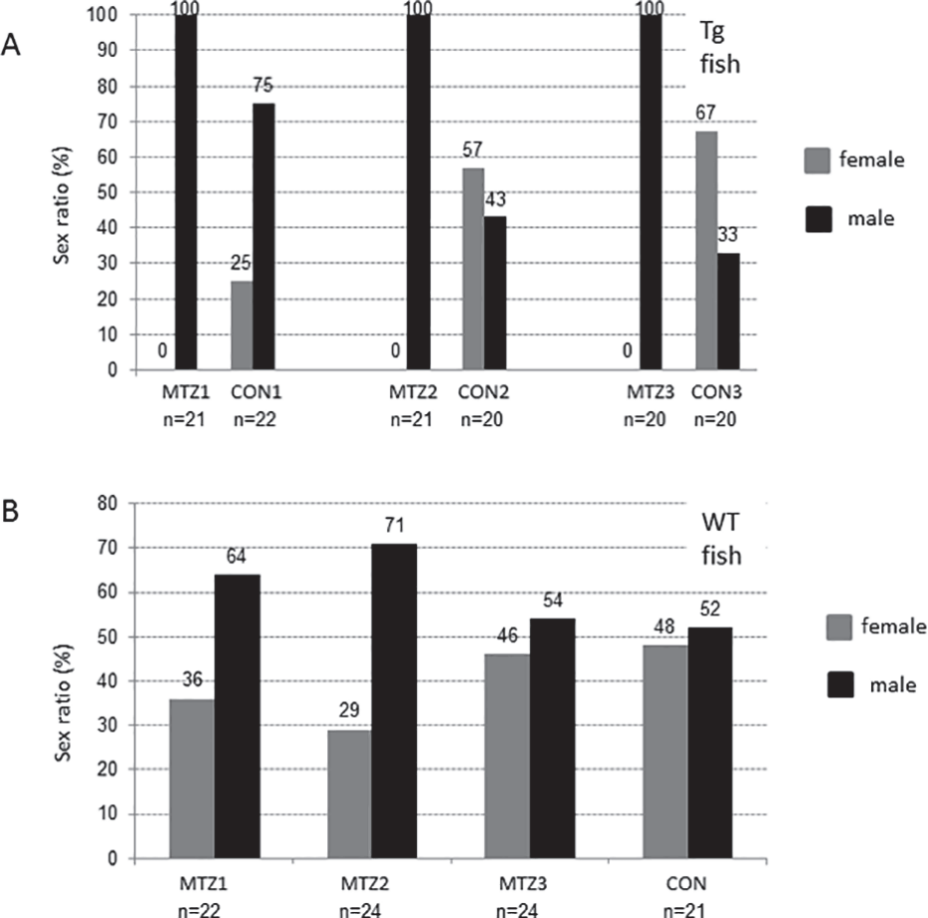
The absence of females developed from MTZ-treated transgenic juveniles. The *dnd^-3’UTR^*:NTR-EGFP transgenic or wild-type sibling control juveniles at 18 dpf were treated with 5 mM MTZ/2% DMSO or 2% DMSO solution for 20 days. The sex ratios of adult fish at 120 dpf were recorded for each of the groups after treatment. A) No female recorded in the groups from 3 independent transgenic lines, line 1 (MTZ1), line 2 (MTZ2) and line 3 (MTZ3) after MTZ-treatment. Various sex ratios of the transgenic fish after DMSO-treatment as the controls were recorded. B) Various sex ratios recorded in the groups of wild-type zebrafish after MTZ-treatments for different periods, 10-day (MTZ1), 20-day (MTZ2), 25-day (MTZ3), or DMSO-treatment for 25 days. The total numbers of fish were labeled under each group.

Each set of Tg(*dnd^-3’UTR^*:NTR-EGFP) larvae at 18 dpf from the same mating were randomly divided equally into two groups. One group of fish were treated with 5 mM MTZ for 20 days until there was no visible EGFP signal in gonads. The other group of fish was treated with vehicle solution for 20 days under the same conditions as the MTZ-treated group. In parallel, a set of wild-type zebrafish was subjected to the same MTZ treatment assays as another control. We found that all MTZ-treated transgenic zebrafish were male (Fig. 6A), after these fish reached sexual maturation. While, the control group of the transgenic fish without MTZ treatment, or MTZ-treated wild-type zebrafish developed into both sexes at various ratios (Fig. 6B). These results were confirmed 3 times independently in different Tg(*dnd^-3’UTR^*:NTR-EGFP) lines. Taken all into account, PGC ablation in Tg*(nd^-3’UTR^*:NTR-EGFP) line mediated by MTZ-NTR technique promoted masculinization in zebrafish.

**Fig 7:**
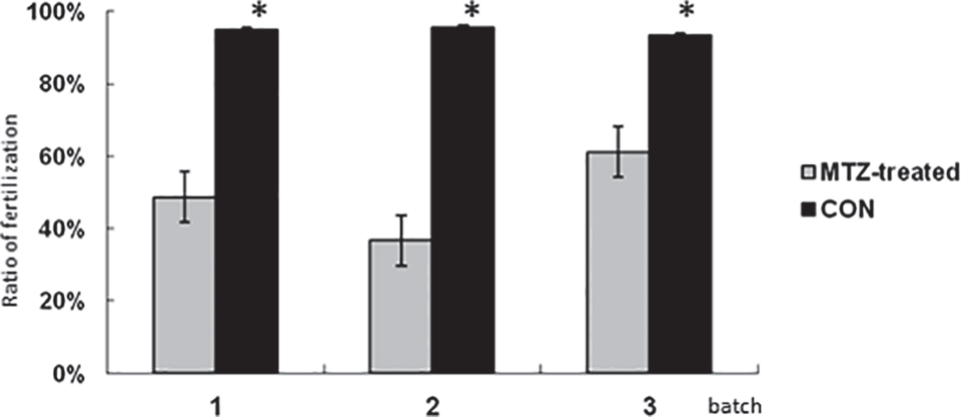
Fertile with impaired fecundity in MTZ-treated transgenic zebrafish males. The fertilization rates of the adult males from 3 independent transgenic lines after MTZ-treatment or DMSO-treatment were recorded. Each group consisted of 10 fishes. The average fertilization rates of the ten fishes of each experiment were measured. The data shown here represents the mean ± the standard error of the mean from three separate experiments.

Unexpectedly, although there was no detectable EGFP signals found in transgenic fish using fluorescence microscopy after the 20-day MTZ treatments, nearly all of the MTZ-treated transgenic males were still fertile. We found that MTZ-treated transgenic males exhibited significantly lower levels of fertilization rates than the control transgenic males in several individual fertilization experiments (Fig. 7). This result suggested that although no EGFP-expressing cells could be detected using fluorescence microscopy following MTZ treatment, the germ cells could still resume when the MTZ-treated transgenic fish reached maturity. Overall, our results provided clear evidence supporting a critical quantity of signals derived from early germ cells that are essential for ovary development in zebrafish.

**Fig 8:**
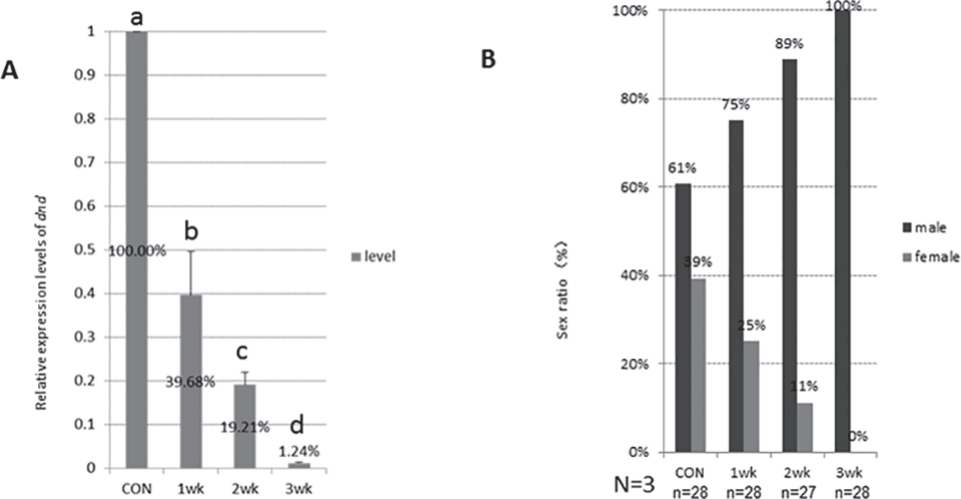
The sufficient number of early germ cells required for the female fate sustain in zebrafish. The *dnd^-3’UTR^*:NTR-EGFP transgenic juvenile siblings were treated with 0.2% DMSO solution from 18 dpf to 39 dpf (con), or 5 mM MTZ/0.2% DMSO solution from 18 dpf to 39 dpf (3-week treatment group), from 25 dpf to 39 dpf (2-week treatment group), and from 32 dpf to 39 dpf stages (1-week treatment group). Total RNA of 5 fish were collected at 39 dpf from each groups. A) relative *dnd* expression levels via quantitative real-time PCR in fish from each groups; B) numbers of the male and female of the rest fish in each groups at their 70 dpf stage. The letters a, b, c, represent the statistically significant difference among groups (*p<0.001*, student T-test). The results represent 3 independent lines.

To further test the essentiality of a sufficient number of the early germ cells for the female fate in zebrafish, Tg(*dnd^-3’UTR^*:NTR-EGFP) fish were then randomly divided into four groups (13 individuals each) at 18 dpf. One of the groups was only treated with the DMSO vehicle solution. The other three groups were treated with 5 mM MTZ for different durations (3 weeks, 2 weeks, and 1 week). These latter treatments began at 18 dpf, 27 dpf, and 32 dpf. All treatments for the four groups ended at 39 dpf. Our quantitative real-time PCR results of *dnd* expression, which represents the relative numbers of the early germ cells, were summaried in Fig. 8A. The significantly decreased level of *dnd* expression was observed after 1 week MTZ treatment. And in 2-, and 3-week treatment groups the levels of *dnd* expression decresed even lower (Fig. 8A). We also found that a dramatically decreased ratio of females happened in 1- and 2-week treatment groups. Even no females can be found in the 3-week treatment groups, in which the level of *dnd* transcripts dropped to less than 5% of that in control fish. Meanwhile, almost 50% of fish in the control group were female (Fig. 8B). These results suggested that a sufficient number of early germ cells are essential for sustaining the female fate in zebrafish.

## Discussion

The tissue distribution of *dnd* mRNA was diverse among species. In mouse, two isoforms of *dnd* gene exist, in which *dnd1-α* is present in early embryos and embryonic gonads, while *dnd1-β* is restricted in the adult testis [21]. In *Xenopus, dnd* transcript (*xdnd*) is detected in embryos until stage 15, after which the expression level decreases to a very low basal level and eventually disappears. *Xdnd* transcript is eventually restricted only to the ovary in adult tissues [22]. In medaka (*Oryzias latipes*), *dnd* is detected at a high level until the morula stage followed by an obvious decrease in later stages. Its adult expression occurs in both sexes and is restricted to germ cells [23]. In zebrafish, essential roles for PGC survival and the maintenance of DND protein have been previously demonstrated [7]. Analyses of zebrafish *dnd* expression have mainly focused on its early PGC-specific patterns [7]. In our present studies, significant levels of *dnd* transcript were observed in early embryos until 48 hpf stage. After 24 dpf, the *dnd* transcript was restricted in PGCs with decreased levels. By 4–6 dpf, the zebrafish *dnd* transcript could not be detected via RT-PCR. At later stages, zebrafish *dnd* transcript could be detected again after 17 dpf. The *dnd* transcript is expressed only in both gonadal tissues, testis and ovary tissue at adult stages (Fig. 4). Several stable germline transmitters were generated using *dnd^-3’UTR^*:NTR-EGFP and *dnd^+3’UTR^*:NTR-EGFP transgenic vectors. These transgenic fish provide unique *in vivo* models for understanding the transcriptional and translational regulation of *dnd*. First, when transgenic females were crossed to nontransgenic males, strong levels of fluorescence protein were expressed ubiquitously and could be observed in the embryos prior to 24 hpf. However, no *ntr-egfp* transcript and fluorescence protein could be observed in the transgenic embryos at 0 hpf from the crosses between the transgenic males and wild-type females (Fig. 2). Interestingly, *ntr-egfp* transcript could not be detected by RT-PCR in the transgenic embryos derived from the transgenic males at 0 hpf but at 5.3 hpf (data not shown). This clearly indicated that the *dnd* transcript and DND protein could be maternally provided in early embryos. The zygotic expression of *dnd* could be initiated at 5.3 hpf during the embryonic stage. Second, during subsequent development from 24 to 48 hpf, weak, ubiquitous and decreasing levels of expression patterns of the fluorescence protein were observed in *dnd^-3’UTR^*:NTR-EGFP positive embryos (Fig. 2G), while a PGC-specific presence of the fluorescence protein with decreasing expression levels were only observed in *dnd^+3’UTR^*:NTR-EGFP embryos (Fig. 2C). This observation supports previous reports on the role of the 3’UTR of *dnd* on PGC-specific expression pattern [24]. Third, in all progenies inheriting the transgene via either the male or female, either *dnd^-3’UTR^*:NTR-EGFP or *dnd^+3’UTR^*:NTR-EGFP transgenic fish, a gonad-specific patterns of the green fluorescent (Fig. 2I) and *ntr-egfp* expression (Fig. 4) could be observed at 17–19 dpf. This expression pattern echoes the presence of the resuming endogenous *dnd* transcript in wild-type zebrafish, which demonstrated the efficacy of the promoter region identified and used in these studies. This observation also suggested that the expressions of *ntr-egfp* and endogenous *dnd* can be resumed in meiotic cells, which was initiated with the appearance of early stage oocyte or onset of sexual differentiation in zebrafish. Finally, the fluorescent protein can be continuously observed in gonadal tissues in adult transgenic fish. These transgenic zebrafish provided ideal opportunities for understanding the DND protein expression in mature gonads. In addition, higher levels of NTR-EGFP could be observed in adult *dnd^-3’UTR^*:NTR-EGFP transgenic fish compared to the adult *dnd^+3’UTR^*:NTR-EGFP transgenic fish (Fig. 3). This demonstrated a gender-independent translational repression role of the 466-bp fragment of the *dnd* 3’ UTR region, which has been previously reported [7].

Transgenic studies can be conducted to analyze gene promoters *in vivo*, and zebrafish are particularly feasible for such analyses. Here, we have also identified a 2032-bp fragment containing the upstream region at positions -2037 to -6 (numbered relative to the transcription start site, Fig. 1), which is capable of recapitulating endogenous *dnd* expression in zebrafish germ cells (Figs 2,3 and 5). Several germ cell-specific promoters have also been previously identified via transgenic studies [25,26,27,28]. We have summarized the features of these reporter expression in transgenic lines (Table 1), the early maternal and zygotic expression patterns of our *dnd^-3’UTR^*:NTR-EGFP transgenic fish are similar with *vas*:EGFP transgenic zebrafish. During our summary period, a report from Wong *et al* was published. An 8.3-kb genomic fragment of the 5’-flanking region of *dnd* (nucleotides 153781 to 162079 in BX890575.23) combined with a 980-bp 3’ UTR region of zebrafish *dnd* gene (nucleotides 172517 to 173493 in BX890575.23) was used in their transgene studies. The authors claimed that a fragment containing a 150-bp region of zebrafish *dnd* spanning the TAG, exon 1, and part of intron 1 (nucleotides 161929 to 162079 in BX890575.23) was essential to drive reporter gene transcription in a gonad-specific expression in their transgenic zebrafish [29]. However, on the basis of our results from the comparison of the expression patterns of the transgenic reporter gene *ntr-egfp* and endogenous *dnd* via *in situ* hybridization experiments (data not shown here), RT-PCR assays (Fig. 4) and fluorescence protein observation (Figs 1–3), a much shorter region of the 5’-flanking region of genomic fragment (2.032-kb fragment at nucleotides 159830 to 161861 in BX890575.23, without the 150-bp critical region identified previously [29], is sufficient and efficient to promote the *ntr-egfp* expression in a gonad-specific manner, which could also be achieved without the presence of the well-recognized the 466-bp fragment of 3’UTR of zebrafish *dnd* [7,24]. According to our observations, this 3’UTR region is probably only critical for the stabilization of the *dnd* mRNA in developing embryos. The difference between our observations and their results regarding the regulatory region may be due to the different lengths of both the 5’ and 3’ genomic DNA regions of *dnd* gene selected for promoter studies, the different 3’-UTR region, or the presence of *Tol2* elements in their vector backbone [29]. However, taking advantage of our fluorescent protein reporter gene, an investigation of the expression patterns of the transgene in our transgenic fish was conducted in a more convenient and comprehensive manner.

**Table 1:** Several transgenic zebrafish for germ cell visualization *in vivo*.

Promoters	Transgenic lines	Earliest zygotic EGFP expression in germ cells (dpf)	Zygotic EGFP expression in adults	References
*β-actin* (*medaka*)	*β-actin*:EGFP	14	female	[25]
zona pellucida	*zp0.5*:EGFP	27–28	female	[26]
RNA helicase vasa	*vas*:EGFP	21–23	female (strong)	[27]
ziwi	*ziwi*:EGFP	7	male and female	[28]
dead end	dnd	?	male and female	[29]
dead end	*dnd*:EGFP	17–18	male and female	our study

Several genetic methods have been reported to specifically ablate cells using different organisms, such as Diphtheria toxin A, Kid/Kis in zebrafish, HSV thymidine kinase/ganciclovir in rodents, Tamoxifen-inducible c-Myc in mouse, Toxic viral protein M2(H37A) in Xenopus embryos. In zebrafish, these two methods were also used for cell ablation in F0 transient transgenic fish. In recent years, the NTR-mediated technique was successfully used in zebrafish to ablate cells of the skin, kidney, testis and ovary in a specific, hereditable and inducible manner [14,15,16,20,30]. Initially, we generated the *dnd*:NTR-EGFP transgenic zebrafish for the purpose of killing germ cells in zebrafish in a specific manner. However, we realized that the efficiency of the NTR-mediated method was not very powerful. The PGC could not be killed using MTZ treatments, when the maternal deposited NTR-EGFP transcript was present during embryonic stages (data not shown). In addition, MTZ treatments were performed at 18 dpf when the zygotic NTR-EGFP expression begins in early germ cells. Our results obtained from the *in situ* cell death assay demonstrated the evident killing effects induced by 1-week MTZ treatments, which were observed in ovary germ cells (Fig. 5). Using a fluorescence microscope, no obvious EGFP signals could be visualized through the fish body wall when treated with MTZ for 20 days. There might be still a small number of early germ cells that survived the MTZ treatments, although no detectable fluorescence was present after the 20-day MTZ treatments. We could still detect low levels of *dnd* expression in 3-week MTZ treated transgenic fish via RT-PCR at 39 dpf stage (Fig. 8A). Besides, we found that there were still fluorescence-positive germ cells later in adult fertile male transgenic fish after the MTZ treatments.

The presence of germ cells plays an important role in sex determination and gonadal development. The zebrafish developed as infertile males when their germ cells were completely depleted [8,31]. In medaka, masculinization also occurs in germ cell-depleted fish [9]. In contrast to these previous studies, early germ cells that were not completely ablate in our present studies. Our MTZ-treated transgenic juveniles all developed as fertile males after a period of MTZ treatment for 20-days at a concentration of 5 mM. Due to the loss of the majority of early germ cells during the sensitive gonadal transition period (Fig. 8), all MTZ-treated transgenic fish treated for 20 days differentiated into males (Fig. 6), with significantly lower levels of fertilization rates compared to those of the control males (Fig. 7). This clearly indicates that it is not the presence, but the sufficiency of the signals derived by the early germ cells is critical for the sex determination of zebrafish during the sex transition stage. Previous studies also shown that the *dnd* morphants after single PGC-transplanted zebrafish developed as males with one normal testis, one thread-like testis, independent of the donor fish (zebrafish, pearl danio, loach, and goldfish). In the discussion section of this previous report, the importance of the quantity of germ cells for zebrafish sex determination has been suggested for the first time [12]. Considering the potential injury of host zebrafish embryos during the single PGC transplantation procedure, our present inducible model provides clear evidence for the essential role of the quantitative signals derived from early germ cell for zebrafish sex fate determination. In 2010, Rodriguez-Mari *et al* reported that in zebrafish that were mutant for *Fanconi anemia* gene, *fancl*, the early oocytes die soon after entry into meiosis, and all of the mutant animals developed as males [4]. This previous report supported our results that a decreased number of early germ cells promoted masculinization in zebrafish. Intriguingly, all our MTZ-treated transgenic zebrafish developed exclusively as fertile males (Figs 6 and 8), which suggested that our inducible treatment procedure may be an effective masculinization method in zebrafish. More recently, Dranow et al reported that the sex-revertion from a portion of the females to sperm-producing males due to the germ cell-depleted when their 5 month old *ziwi*:CFP-NTR adult female transgenic zebrafish were treated with MTZ [17]. One of the main differences between their reports and ours is the period for the MTZ treatments. Therefore, our current studies indicate that the presence of the sufficient signals derived from the early germ cells at before 40 dpf stage is critical for the sex determination of zebrafish.

In teleosts, sex determination mechanisms were much more diverse than the mammalian XY/XY-system. The zebrafish immature gonads “juvenile ovaries” can be guided to develop into testis or ovaries between 21–42 dpf, while sexually maturity was achieved after approximately three months. This process are differed slightly among individuals and breeding conditions [32]. However, little is known regarding the precise mechanism in zebrafish sex determination. In our present studies, we identified a germ cell-specific promoter of the *dnd* gene. With the combination of the *dnd^-3’UTR^*:NTR-EGFP and *dnd^+3’UTR^*:NTR-EGFP transgenic lines, we were able to demonstrate the regulatory function of the 3’UTR in DND translation. In addition, the analyses of the *dnd*:NTR-EGFP transgenic modelswith the MTZ treatments also demonstrate that the quantity, rather than the presence, of the signals derived from the early germ cells plays an essential role in zebrafish sex fate determination. This study also provides a potential model for the development of a sex control technique in zebrafish husbandry and research.
